# The associations between infertility-related stress, family adaptability and family cohesion in infertile couples

**DOI:** 10.1038/s41598-021-03715-9

**Published:** 2021-12-20

**Authors:** Anjiang Lei, Huaxuan You, Biru Luo, Jianhua Ren

**Affiliations:** 1 Department of Obstetrics and Gynecology Nursing, West China Second University Hospital, Sichuan University/West China School of Nursing, Sichuan University, Chengdu, Sichuan China; 2 Key Laboratory of Birth Defects and Related Diseases of Women and Children (Sichuan University), Ministry of Education, Chengdu, Sichuan China

**Keywords:** Health care, Medical research, Risk factors

## Abstract

To explore the association between infertility-related stress, family adaptability and family cohesion in infertile couples and the determinants of infertility-related stress in infertile couples. Fertility Problem Inventory (FPI) and Family Adaptability and Cohesion Evaluation Scales (FACESII-CV) were used to measure the infertility-related stress and family adaptability and cohesion of infertile couples. T-test, ANOVA and multiple comparisons (LSD) were conducted to compare the FPI scores of different demographic characteristics subgroups. Stepwise multivariate linear regression was used to explore the determinants of infertility-related stress. Women had greater global stress than men (*P* < 0.001). Women scored higher on desired family adaptability, cohesion dissatisfaction and adaptive dissatisfaction than men (*P* = 0.039, *P* = 0.036, *P* = 0.008). FPI scores were higher in men and women who lived in rural (*P* < 0.001, *P* < 0.001). Family cohesion and education level was negatively correlated with infertility-related stress in men. Family adaptability and education level was negatively correlated with infertility-related stress in women. Healthcare providers should pay more attention and give more support to infertile couples who lived in rural or with low education level, and provide easier medical accessing for them. Moreover, healthcare providers should value more the family function and family support in intervention of reducing infertility-related stress.

## Introduction

Infertility has been recognized as an internationally relevant social and public health issue, which affects quality of life, mental health, marital satisfaction and relationship with family^1–3^. Globally, the incidence of infertility among women aged 25 to 44 years ranges from 3.5 to 16.7% in developed countries, 6.9% to 9.3% in developing countries^4^. The age-standardized prevalence rate of infertility increased by 0.37% per year for females and 0.29% per year for males from 1990 to 2017^5^. In China, the incidence of infertility was up to 25% among couples of reproductive age^6^.

The infertility produces infertility-related stress in both members of infertile couples^7^. Infertility-related stress has adverse effect on couples’ quality of life and relationship with family^8^. Some studies showed that the level of infertility related-stress was negatively related to IVF success rate and positive pregnancy outcome after IVF^9–11^. Another studies reported that IVF failure predicted psychological distress, but psychological stress was not related the IVF failure^12,13^. Most of studies reported that women seem to experience more stress than their partner because of childlessness^10,14,15^. And the stress level of infertility couples as associated with psychological distress level of their partner, and stress of both partners was associated with adverse IVF outcome^16^. In traditional beliefs of Chinese, childlessness is unfilial. Culture difference might cause different social pressure and mental stress for Chinese papulation^17^. Although the negative effects of infertility-related stress have been reported in the literature, its influencing factors of Chinese population is necessary to investigate^7,15,18^. In China, personal interdependence was emphasized and most people tend to give priority to the needs of family members over their own^19^. Therefore, there might be associations between infertility-related stress and family function. Previous studies showed the age, gender, employment status, economic situation and education level were associated with mental health of infertile couples^17,20,21^, so in order to explore the determinants of infertility-related stress in infertile couples, the sociodemographic characteristics were recorded and taken as independent variables.

Family is the individuals’ sociocultural environment which can affect members’ health^22^. Family function is associated with members’ mental health and health behaviors^23^. Adaptability and cohesion are dimensions of family functioning. Family adaptability is the ability to change roles in response to problems or stressful events. Family cohesion is understood as the emotional bond between family members^24^. Previous studies showed that higher family cohesion and adaptability resulted in lower degree of depression in terminally ill cancer patients^22^. Family cohesion and adaptability were reported strongly related to psychological adjustment of adolescent cancer survivors^25^. In the study of Hidalgo et al., the concepts of cohesion and adaptability did not differentiate infertile couples from fertile ones^26^. And we noticed the small sample size of infertile group. In Chinese literature, we found some studies focused on the infertile women^27,28^. There is little research focus on the family cohesion and adaptability of Chinese infertile couples. Moreover, the association between infertility related stress and family cohesion and adaptability in infertile couples is still unclear.

This study was aimed to measure the infertility-related stress and family adaptability and cohesion of infertility couples, and explored the association between them. Stepwise multivariate linear regression was conducted to find the protective and risk factors of fertility-related stress. The hypothesis was proposed: Family adaptability and family cohesion were negatively correlated with infertility-related stress of infertile couples.

## Methods

### Study design

All methods were performed in accordance with the Declaration of Helsinki. A cross-sectional survey was conducted on infertile couples recruited from West China Second University Hospital of Sichuan University, which is a women and children’s medical center in western China that serves over 5 provinces. Patients who met the inclusion criteria were selected as subjects.

### Participants

Participants were eligible if they: (1) were diagnosed with infertility; (2) were volunteered to participate in the study both themselves and their partner; (3) had no known history of auditory, language, or cognitive problems. Participants were excluded if they: (1) had one child; (2) had other severe organic disease or mental illness.

### Sample size

According to Kendall’s experience and methods, sample size can be 5 to 10 times the number of independent variables^29^. Our sample size was 10 times the number of independent variables. Considering the unqualified questionnaire, sample size was increased by 10% to 506 couples.

### Measurement

The demographic data and clinical data were collected through a self-designed questionnaire, which included socio-demographic information such as age, nationality, education level, place of residence, occupation, family per capita monthly income and clinical information such as type of infertility and the duration of infertility.

#### Fertility Problem Inventory (FPI)

Fertility Problem Inventory (FPI) was developed by Newton et al. in 1999^30^. The FPI is a 46-item self-rating scale assessing level of infertility-related stress. All items are scored using a 6-point Likert scale ranging from 1 (I do not agree) to 6 (I totally agree). Global stress is calculated by summing all five subscale scores, and item 1, 4, 7, 9, 12, 15, 18, 20, 23, 25, 28, 31, 33, 35, 38, 41, 44, 46 are reverse scoring. The minimum score is 46, and the maximum score is 276. Higher scores indicate higher infertility-related stress. It has been translated into several languages and has been widely used in a range of clinical settings. This questionnaire included 5 dimensions: social concerns (10 items), relationship concerns (10 items), need for parenthood (10 items), rejection of childfree lifestyle (8 items) and sexual concerns (8 items). The test–retest reliability of the FPI was 0.83 (female), 0.84 (male), and the Cronbach’s α coefficient was 0.77–0.93. M-FPI (Mandarin version of FPI) was reliable and valid for Chinese infertile couples, and the Cronbach’s α coefficient was 0.81^31^. In our pilot study, 30 female Chinese infertility couples were pre-surveyed by FPI, the Cronbach's α coefficient was 0.927. Confirmatory factor analysis was conducted, the standardized factor loading values ranged from 0.416 to 0.722. Satisfied convergent validity and discriminant validity were confirmed in previous study^31^. M-FPI was verified as a reliable and valid instrument and can be used effectively with infertile Chinese couples.

#### Family Adaptability and Cohesion Evaluation Scales (FACES II-CV)

Family Adaptability and Cohesion Evaluation Scales (FACES II) were developed by Olson in 1982 and were translated into Mandarin (FACES II-CV) by Fei Lipeng in 1991. FACES II-CV is a self-rating scale with 30 items on a 5-point Likert scale that measures perceptions of family adaptability (14 items) and cohesion (16 items)^32^. All items are scored using a 5-point Likert scale ranging from 1 (almost never) to 5 (almost always). Total score of Family cohesion = 36 + Item1 + Item5 + Item7 + Item11 + Item13 + Item15 + Item17 + Item21 + Item23 + Item25 + Item27 + Item30 − Item3 − Item9 − Item19 − Item29. Total score of family adaptability = 12 + Item2 + Item4 + Item6 + Item8 + Item10 + Item12 + Item14 + Item16 + Item18 + Item20 + Item22 + Item26 − Item24 − Item28. Higher scores indicate higher family adaptability and cohesion. Each participant needed to answer FACES II-CV twice, one time for actual feeling and another time for desired ideal of family situation. The scores difference of two times indicated dissatisfaction with family cohesion and adaptability. The greater the difference, the greater the degree of dissatisfaction. The test–retest reliability was 0.84 and 0.54, Cronbach’s α coefficient was 0.944^33^. In this study, 30 female infertility patients were pre-surveyed by FACES II-CV, the Cronbach’s α coefficient was 0.901. Confirmatory factor analysis was conducted, the standardized factor loading values ranged from 0.451 to 0.726. Satisfied convergent validity and discriminant validity were confirmed in previous study^34^. FACES II-CV was verified as a reliable and valid instrument and can be appropriate for use in China.

### Data analysis

SPSS 21.0 (SPSS Inc, Chicago, IL) was used for statistical analysis. Means (M), standard deviations (SD), number (N) and percentage (%) were used to describe the demographic and clinical variables. T-tests were performed to evaluate the mean differences between men and women regarding their infertility-related stress. T-test, ANOVA and multiple comparisons (LSD) were conducted to compare the FPI scores of different demographic characteristics subgroups. Pearson correlation was conducted to explore the correlation between infertility-related stress and family cohesion, family adaptability. Predictor variables (*P* ≤ 0.1) were entered into the Stepwise linear regression. Stepwise multivariate linear regression was used to explore the influence factors of infertility-related stress. In all analyses, a *P* value of < 0.05 indicated statistical significance.

### Ethics approval and consent to participate

The study was approved by the ethics committee of the West China Second University Hospital of Sichuan University. Written informed consent was provided by the participants before the investigation.

## Results

### Demographic characteristics

A total of 540 infertile couples were recruited in this study, but 32 of these were excluded because of more than 10% of the items were not completed. 508 infertile couples’ data included in statistical analyses. Participants’ characteristics are shown in Table 1.

**Table 1:** Characteristics of infertile couples (N = 508).

Variable	Men Mean ± SD/N (%)	Women Mean ± SD/N (%)
Age	31.06 ± 4.18	29.32 ± 3.90
**Nationality**		
Han	491 (96.65)	481 (94.69)
Minority	17 (3.35)	27 (5.31)
**Education level**		
Middle school or below	59 (11.61)	67 (13.19)
High school	119 (23.43)	117 (23.03)
Some college	135 (26.57)	139 (27.36)
College	164 (32.28)	166 (32.68)
Master or more	31 (6.10)	19 (3.74)
**Place of residence**		
Urban	346 (68.11)	340 (66.93)
Country	162 (31.89)	168 (33.07)
**Occupation**		
Professional	133 (26.18)	122 (24.02)
Farmer	24 (4.72)	16 (3.15)
Administrative	56 (11.03)	47 (9.25)
Freelance	101 (19.88)	64 (12.60)
Unemployed	11 (2.17)	81 (15.94)
Others	183 (36.02)	178 (35.04)
**Family per capita monthly income**		
< 3000	53 (10.43)	53 (10.43)
3000–5999	155 (30.51)	155 (30.51)
6000–8999	117 (23.03)	117 (23.03)
> 9000	183 (36.02)	183 (36.02)
**Type of infertility**		
Primary	303 (59.65)	303 (59.65)
Second	205 (40.35)	205 (40.35)
Duration of infertility (years)	3.45 ± 2.08	3.10 ± 2.73

### The FPI scores of infertile couples

The scores of infertility-related stress among infertile couples differed by gender. Women had greater global stress than men (*P* < 0.001), and had greater specific stress in terms of social concerns, relationship concerns, need for parenthood, and sexual concerns than men (*P* < 0.001, *P* = 0.009, *P* = 0.004, *P* < 0.001) (Table 2).

**Table 2:** The FPI scores of infertile couples (N = 508).

	Men Mean ± SD	Women Mean ± SD	t	*P*
Social concerns	26.01 ± 7.76	28.60 ± 8.31	− 5.45	** < 0.001**
Relationship concerns	28.00 ± 6.48	29.02 ± 6.91	− 2.63	**0.009**
Need for parenthood	40.41 ± 9.22	42.06 ± 8.96	− 2.24	**0.004**
Rejection of childfree lifestyle	28.37 ± 6.54	27.89 ± 6.79	1.37	0.170
Sexual concerns	17.77 ± 6.02	19.28 ± 6.96	− 4.50	** < 0.001**
Global stress	140.56 ± 26.47	147.02 ± 28.44	− 3.94	** < 0.001**

Significant values are given in bold.

### The scores of family cohesion and adaptability in infertile couples

The difference of desired family adaptability between women and man was significant. Women scored higher on desired family adaptability than men (*P* = 0.039). Women scored higher on cohesion dissatisfaction than men (*P* = 0.036). Women have higher adaptive dissatisfaction than men (*P* = 0.008) (Table 3).

**Table 3:** The scores for family adaptability and cohesion in infertile couples (N = 508).

	Men Mean ± SD	Women Mean ± SD	t	*P*
Actual cohesion	71.25 ± 9.20	70.99 ± 9.92	0.44	0.661
Desired cohesion	75.67 ± 9.70	76.60 ± 9.79	− 1.52	0.129
Actual adaptability	49.00 ± 4.90	48.78 ± 8.37	0.44	0.663
Desired adaptability	54.11 ± 8.64	55.24 ± 8.74	− 2.07	**0.039**
Cohesion dissatisfaction	4.42 ± 7.96	5.61 ± 8.74	− 2.27	**0.036**
Adaptive dissatisfaction	5.11 ± 7.47	6.46 ± 8.74	− 2.65	**0.008**

Significant values are given in bold.

### FPI scores of different demographic characteristics subgroups

T-test, ANOVA and multiple comparisons (LSD) were conducted to compare the FPI scores of different demographic characteristics subgroups. In the results, FPI scores were lower in men and women who lived in urban (*P* < 0.001) (Table 4). In multiple comparisons, the FPI scores of men with under college education were higher than those with college or above, the FPI scores of women with high school education or below were higher than those with some college education or above (Tables 5, 6).

**Table 4:** Comparisons of FPI scores of different demographic characteristics subgroups (N = 508).

Group	Men	t/F	*P*	Women	t/F	*P*
**Residence**^t^						
Urban	137.57 ± 26.35	− 3.682	** < 0.001**	144.19 ± 29.13	− 3.811	** < 0.001**
Rural	146.86 ± 26.86			154.35 ± 26.40		
**Education level**^A^						
Middle school or below	145.63 ± 25.40	6.680	** < 0.001**	154.99 ± 27.29	8.032	** < 0.001**
High school	145.74 ± 23.82			157.04 ± 25.65		
Some college	144.49 ± 27.34			144.79 ± 29.65		
College	133.92 ± 27.82			141.92 ± 28.02		
Master or more	128.65 ± 23.17			132.37 ± 28.24		

^t^t-test.

^A^ANOVA.

Significant values are given in bold.

**Table 5:** Multiple comparisons of FPI scores of men with different education level (N = 508).

Educational level (I)	Educational level (J)	Mean difference (I–J)	Std. error	*P*
College	Middle school or below	− 11.706	3.986	**0.003**
	High school	− 11.819	3.162	**0.000**
	Some college	− 10.568	3.051	**0.001**
	Master or more	5.276	5.142	0.305
Master or more	Middle school or below	− 16.982	5.824	**0.004**
	High school	− 17.094	5.294	**0.001**
	Some college	− 15.844	5.229	**0.003**
	College	− 5.276	5.142	0.305

Significant values are given in bold.

**Table 6:** Multiple comparisons of FPI scores of women with different education level (N = 508).

Educational level (I)	Educational level (J)	Mean difference (I–J)	Std. error	*P*
Middle school or below	High school	− 2.058	4.270	0.630
	Some college	10.194	4.145	**0.014**
	College	13.069	4.034	**0.001**
	Master or more	22.617	7.244	**0.002**
High school	Middle school or below	2.058	4.270	0.630
	Some college	12.251	3.497	**0.000**
	College	15.127	3.364	**0.000**
	Master or more	24.674	6.893	**0.000**

Significant values are given in bold.

### Multiple linear regression analysis of infertility-related stress in infertile couples

Pearson correlation between FPI and actual family cohesion, actual family adaptability are shown in Table 7. Predictor variables (*P* ≤ 0.1) were entered into the Stepwise linear regression. The contributions of all significant factors in the final model are shown in Table 8. Actual family cohesion and education level was negatively correlated with infertility-related stress in men, and explained 15.5% of its variance. Actual family adaptability and education level was negatively correlated with infertility-related stress in women, and explained 9.2% of its variance.

**Table 7:** Pearson correlation analysis between infertility-related stress and family cohesion, family adaptability.

	Men Total score of FPI	Women Total score of FPI
Actual cohesion	− 0.237 (**< 0.001**)	− 0.381 (**< 0.001**)
Actual adaptability	− 0.254 (**< 0.001**)	− 0.295 (**< 0.001**)

Significant values are given in bold.

**Table 8:** Multiple regression analysis of infertility-related stress in infertile couples.

Men				Women			
	*β*	*P*	Adj. R^2^		*β*	*P*	Adj. R^2^
Actual cohesion	− 0.826	** < 0.001**	0.155	Actual cohesion	− 0.091	0.198	0.092
Actual adaptability	− 0.110	0.096		Actual adaptability	− 0.744	** < 0.001**	
Residence	0.076	0.116		Residence	0.072	0.149	
Education level	− 3.713	** < 0.001**		Education level	− 4.559	** < 0.001**	

Significant values are given in bold.

## Discussion

Women of infertile couples were showed greater infertility-related global stress and greater specific stress in terms of social concerns, relationship concern, need for parenthood, and sexual concerns than men. This indicated that women had more negative experiences with infertility than men in most of the domains. This was consistent with previous studies conducted worldwide. The result of Ying et al. indicated that women had more negative experiences in the domains of physical stressors, existential stressors, and emotional stressors than men of infertile couples^35^. The research of Cserepes et al. showed that infertility-related global stress, infertility-related social concerns had more intensive effect on women than on men^15^. The fact that women of infertile couples are more stressful than men may be caused by their gender roles and sex-role identification^18,36^. Traditionally, motherhood has a more convergent correlation with feminine roles than fatherhood has with masculinity^15^. Compared to the research of Peterson et al. focusing on Caucasian population, the infertility-related stress level of Chinese couples was higher than Caucasian^37^. Moreover, the infertility-related stress level of Hungarian couples was reported lower than Chinese couples^15^. We inferred that might be due to the traditional beliefs and culture difference. In traditional beliefs of Chinese, childlessness is unfilial, which might cause higher social pressure and mental stress for Chinese papulation^17^. Previous study showed that partner support was in favor of coping with infertility-related distress^35^. Therefore, future study may focus on enhancing a sense of partnership and partner support among infertile couples to help them to cope with infertility-related stress. Culture difference should be considered in psychological support for infertile couples.

Family adaptability is the extent flexibility of a family when problems or changes occur. Family cohesion is the degree to which family members experience an emotional bond between each other^22^. In this study, the scores of desired adaptability and adaptive dissatisfaction of women were higher than men of infertile couples, which indicated that women of infertile couples need more communication with their partners or other family members, and hope every members in their family could participate in the decision-making. Moreover, women were found higher cohesion dissatisfaction than men of infertile couples, which indicated that women were less satisfied with the degree of emotional connection between their partners. The reason might be the women's emotional richness and higher social pressure^38^. Compared to the Chinese norm, infertile couples had higher score of actual and desired family cohesion, but lower score of actual and desired family adaptability, which was similar to previous study^27,28^. We inferred that might be the infertile couples developed tight emotional bond when they received treatment. But the long treatment cycle and financial burden might reduce the family adaptability. Therefore, healthcare providers should encourage men of infertile couples to communicate with their partners and help the infertile couples to develop an emotional bond. Future research could develop tailored intervention to help infertile couples to improve family adaptability.

The results of one-way ANOVA showed that the FPI scores of infertile couples differed significantly depending on where they lived. The infertile couples who lived in rural had higher FPI scores than those in urban (*P* < 0.001). This implied that infertile couples who lived in rural had more negative experiences and infertility-related stress than those who lived in urban. This is similar with previous studies, which reported that urban older adults had relatively higher cognitive ability and better psychological health than their rural counterparts in China^39,40^. As reported, shortages of health providers in rural areas and geographic isolation make accessing for mental healthcare more difficult for rural populations^41^. Therefore, we inferred that infertile couples who lived in rural with greater infertility-related stress because of insufficient information and medical resource. In addition, people live in rural areas may have more traditional concepts about infertility, which may increase their infertility-related stress. China is a developing country, there are huge gaps in terms of economy, social security and health services between urban and rural areas^39,40^. Healthcare providers should pay more attention and support infertile couples who lived in rural, and help them to face the illness and reduce the infertility-related stress.

The multiple comparisons showed that education level were significant determinants of the FPI scores of infertile couples (*P* < 0.001). The FPI scores of men with under college education were higher than those with college or above, the FPI scores of women with high school education or below were higher than those with some college education or above. This is consistent with previous study conducted in China^42^. Compared to individuals with higher education level, those educated below high school graduation had higher risk of mental disorders^43–45^. We inferred that infertile couples with higher education level may be more likely to acquire knowledge about infertility, or have a better way to seek professional assistance. Therefore, healthcare providers should pay more attention to infertile couples who with lower education level and provide easier accessing to medical resources.

In multiple linear regression, family cohesion and education level was found negatively correlated with infertility-related stress in men. Family adaptability and education level was negatively associated with infertility-related stress in women. This result may be attributed to that the better family function helps to reduce infertility-related stress. This was similar to previous study, which reported that family function is linked with stress through health behaviors, and better family function with better mental health^22,23,46^. Moreover, better family function was associated with better control of chronic diseases and higher quality of life^47,48^. Healthcare providers should value the family support of infertile couples especially who with lower education level. In order to release the infertility-related stress, future studies could explore the effective interventions on family function promotion.

It should be noted that this study contains several limitations. First, there is a possibility of selection bias because only patients admitted to a hospital were selected. Second, the sample may not be representative of all infertile couples in China, as they were recruited from one hospital in western China. Third, most of infertile couples had a female factor diagnosis, so the infertile couples with male factor diagnosis were underrepresented.

## Conclusion

We found infertility-related stress of women were greater than men. Women of infertile couples desired higher family adaptability, but most of them were not satisfied with their actual family adaptability and cohesion. The infertile couples who lived in rural, had greater infertility-related stress than those in urban. Infertile couples with higher education level scored lower on infertility-related stress. Family cohesion and education level was negatively correlated with infertility-related stress in men. Family adaptability and education level was negatively correlated with infertility-related stress in women.

## References

[1] Ben Shlomo S, Pascal M, Taubman Ben-Ari O, Azuri Y, Horowtz E (2017). Life satisfaction of women in early stages of fertility treatment. Women Health.

[2] Palomba S (2018). Lifestyle and fertility: The influence of stress and quality of life on female fertility. Reprod. Biol. Endocrinol..

[3] Inhorn M, Patrizio P (2015). Infertility around the globe: New thinking on gender, reproductive technologies and global movements in the 21st century. Hum. Reprod. Update.

[4] Mascarenhas M (2013). Trends in primary and secondary infertility prevalence since 1990: A systematic analysis of demographic and reproductive health surveys. Lancet.

[5] Zhou Z (2018). Epidemiology of infertility in China: A population-based study. BJOG.

[6] Sun H (2019). Global, regional, and national prevalence and disability-adjusted life-years for infertility in 195 countries and territories, 1990–2017: Results from a global burden of disease study, 2017. Aging.

[7] Casu G (2018). Spirituality, infertility-related stress, and quality of life in Brazilian infertile couples: Analysis using the actor-partner interdependence mediation model. Res. Nurs. Health.

[8] Mancuso A (2020). Infertility and health-related quality of life in United States women Veterans. J. Womens Health.

[9] Aimagambetova G (2020). The effect of psychological distress on IVF outcomes: Reality or speculations?. PLoS ONE.

[10] Donarelli Z (2012). Are attachment dimensions associated with infertility-related stress in couples undergoing their first IVF treatment? A study on the individual and cross-partner effect. Hum. Reprod..

[11] Zhou F, Cai Y, Dong Y (2019). Stress increases the risk of pregnancy failure in couples undergoing IVF. Stress.

[12] Bapayeva G (2021). The effect of stress, anxiety and depression on in vitro fertilization outcome in Kazakhstani Public clinical setting: A cross-sectional study. J. Clin. Med..

[13] Miller N (2019). Does stress affect IVF outcomes? A prospective study of physiological and psychological stress in women undergoing IVF. Reprod. Biomed. Online.

[14] Kim J, Shin H, Yun E (2018). A dyadic approach to infertility stress, marital adjustment, and depression on quality of life in infertile couples. J. Holist. Nurs..

[15] Cserepes RE, Kollár J, Sápy T, Wischmann T, Bugán A (2013). Effects of gender roles, child wish motives, subjective well-being, and marital adjustment on infertility-related stress: A preliminary study with a Hungarian sample of involuntary childless men and women. Arch. Gynecol. Obstet..

[16] Haimovici F (2018). Stress, anxiety, and depression of both partners in infertile couples are associated with cytokine levels and adverse IVF outcome. Am. J. Reprod. Immunol..

[17] Li X, Wang K, Huo Y, Zhou M (2018). The effect of infertility-related stress on Chinese infertile females’ mental health: The moderating role of marital adjustment. PsyCh J..

[18] Grunberg P, Miner S, Zelkowitz P (2020). Infertility and perceived stress: The role of identity concern in treatment-seeking men and women. Hum. Fertil..

[19] Li X, Zhao N, Zhou M, Liu J, Zhang J (2016). Ren Qin and interpersonal trust: The moderating role of Guanxi type and tasks domain. Psychol. Explor..

[20] Zurlo M, Cattaneo Della Volta M, Vallone F (2020). Infertility-related stress and psychological health outcomes in infertile couples undergoing medical treatments: Testing a multi-dimensional model. J. Clin. Psychol. Med. Settings.

[21] Yilmaz T, Yazici S, Benli T (2020). Factors associated with infertility distress of infertile women: A cross-sectional study. J. Psychosom. Obstet. Gynaecol..

[22] Park Y (2018). The influence of family adaptability and cohesion on anxiety and depression of terminally ill cancer patients. Support. Care Cancer.

[23] Cheng Y (2017). The effects of family structure and function on mental health during China's transition: A cross-sectional analysis. BMC Fam. Pract..

[24] Olson D, Russell C, Sprenkle D (1983). Circumplex model of marital and family systems: VI. Theoretical update. Fam. Process.

[25] Zhou Y (2020). The mechanism of family adaptability and cohesion in suicidal ideation among Chinese cancer patients. J. Psychosoc. Oncol..

[26] Hidalgo M, Caleffi L, Baron A, Mattana E, Chaves M (2004). Cohesion and adaptability among individuals under treatment for infertility. Psychol. Rep..

[27] Song D, Wang D, Lu H (2010). Correlation between anxiety and family resolve and adaptability among infertile women treated with IVF-ET before taking ovum. J. Nurs. Sci..

[28] Shen, S. *The Effects of Family Cohesion and Adaptation on Resilience and Medical Coping Modes Questionnaire in The Female Infertility Patients.* Master thesis, Anhui Medical University (2017).

[29] Chen B, Kang D (2014). Clinical Epidemiology.

[30] Newton C, Sherrard W, Glavac I (1999). The Fertility Problem Inventory: Measuring perceived infertility-related stress. Fertil. Steril..

[31] Peng T, Coates R, Merriman G, Zhao Y, Maycock B (2011). Testing the psychometric properties of Mandarin version of the Fertility Problem Inventory (M-FPI) in an infertile Chinese sample. J. Psychosom. Obstet. Gynaecol..

[32] Li M (2021). Perceived family adaptability and cohesion and depressive symptoms: A comparison of adolescents and parents during COVID-19 pandemic. J. Affect. Disord..

[33] Liu, Y. *A Study on the Correction Between Psychological Resilience, Family Adaptability and Cohesion and Benefit Finding Among Female Infertile Patients.* Master thesis, Jinzhou Medical University (2020).

[34] Phillips M, West C, Shen Q, Zheng Y (1998). Comparison of schizophrenic patients' families and normal families in China, using Chinese versions of FACES-II and the Family Environment Scales. Fam. Process.

[35] Ying L, Wu L, Loke A (2015). Gender differences in experiences with and adjustments to infertility: A literature review. Int. J. Nurs. Stud..

[36] Berg B, Wilson J, Weingartner P (1991). Psychological sequelae of infertility treatment: The role of gender and sex-role identification. Soc. Sci. Med..

[37] Peterson BD, Newton CR, Rosen KH (2003). Examining congruence between partners' perceived infertility-related stress and its relationship to marital adjustment and depression in infertile couples. Fam. Process.

[38] Kim Y, Kim S, Joh J (2015). Family adaptability and cohesion in families consisting of Asian immigrant women living in South Korea: A 3-year longitudinal study. Asia-Pac. Psychiatry.

[39] Guo Q, Bai X, Feng N (2018). Social participation and depressive symptoms among Chinese older adults: A study on rural-urban differences. J. Affect. Disord..

[40] Sun J, Lyu S (2020). Social participation and urban-rural disparity in mental health among older adults in China. J. Affect. Disord..

[41] Andrilla C, Garberson L, Patterson D, Quigley T, Larson E (2020). Comparing the health workforce provider mix and the distance travelled for mental health services by rural and urban medicare beneficiaries. J. Rural. Health.

[42] Luo L, Shi L (2018). Impact of infertility-related psychological stress on the quality of life of azoospermia patients. Natl. J. Androl..

[43] Kuklová M (2021). Educational inequalities in mental disorders in the Czech Republic: Data from CZEch Mental health Study (CZEMS). Soc. Psychiatry Psychiatr. Epidemiol..

[44] Niemeyer H, Bieda A, Michalak J, Schneider S, Margraf J (2019). Education and mental health: Do psychosocial resources matter?. SSM Popul. Health.

[45] Kurtze N, Eikemo T, Kamphuis C (2013). Educational inequalities in general and mental health: Differential contribution of physical activity, smoking, alcohol consumption and diet. Eur. J. Public Health.

[46] Yeom H, Lee J (2020). Gender difference in the relationship among family function, health behavior, and stress in midlife. Int. J. Aging Hum. Dev..

[47] Strizich G (2016). Glycemic control, cognitive function, and family support among middle-aged and older Hispanics with diabetes: The Hispanic Community Health Study/Study of Latinos. Diabetes Res. Clin. Pract..

[48] Rosalini M (2019). Quality of life, cohesion and adaptability in beneficiary families of the “Bolsa Família” Program. Cien. Saude Colet..

